# Extracellular vesicles carry cellulases in the industrial fungus *Trichoderma reesei*

**DOI:** 10.1186/s13068-019-1487-7

**Published:** 2019-06-15

**Authors:** Renato Graciano de Paula, Amanda Cristina Campos Antoniêto, Karoline Maria Vieira Nogueira, Liliane Fraga Costa Ribeiro, Marina Campos Rocha, Iran Malavazi, Fausto Almeida, Roberto Nascimento Silva

**Affiliations:** 1 Department of Biochemistry and Immunology, Ribeirao Preto Medical School, University of Sao Paulo, Ribeirao Preto, São Paulo, 14049-900 Brazil; 2 Departamento de Genética e Evolução, Centro de Ciências Biológicas e da Saúde, Universidade Federal de São Carlos, São Paulo, Brazil

**Keywords:** *Trichoderma reesei*, Extracellular vesicles, Cellulases, Secretion, Proteome

## Abstract

**Background:**

*Trichoderma reesei* is the most important industrial producer of lignocellulolytic enzymes. These enzymes play an important role in biomass degradation leading to novel applications of this fungus in the biotechnology industry, specifically biofuel production. The secretory pathway of fungi is responsible for transporting proteins addressed to different cellular locations involving some cellular endomembrane systems. Although protein secretion is an extremely efficient process in *T. reesei*, the mechanisms underlying protein secretion have remained largely uncharacterized in this organism.

**Results:**

Here, we report for the first time the isolation and characterization of *T. reesei* extracellular vesicles (EVs). Using proteomic analysis under cellulose culture condition, we have confidently identified 188 vesicular proteins belonging to different functional categories. Also, we characterized EVs production using transmission electron microscopy in combination with light scattering analysis. Biochemical assays revealed that *T. reesei* extracellular vesicles have an enrichment of filter paper (FPase) and β-glucosidase activities in purified vesicles from 24, 72 and 96, and 72 and 96 h, respectively. Furthermore, our results showed that there is a slight enrichment of small RNAs inside the vesicles after 96 h and 120 h, and presence of hsp proteins inside the vesicles purified from *T. reesei* grown in the presence of cellulose.

**Conclusions:**

This work points to important insights into a better understanding of the cellular mechanisms underlying the regulation of cellulolytic enzyme secretion in this fungus.

**Electronic supplementary material:**

The online version of this article (10.1186/s13068-019-1487-7) contains supplementary material, which is available to authorized users.

## Background

*Trichoderma reesei* is one of the main producers of cellulolytic and xylanolytic enzymes, being the most studied fungus involved with lignocellulosic degradation [1–4]. It has the ability to colonize a wide range of habitats and is able to fully break down different types of biomass by secreting a range of enzymes and metabolites [5–9]. In *T. reesei*, the gene expression and secretion of enzymes are directly dependent on the different chemical signals produced from diverse substrates. This feature provides the ability to grow in a wide range of carbon sources and the production a variable range of cellulases [10].

The natural ability to produce and secrete large amounts of protein makes *T. reesei* an interesting host for production of heterologous proteins used in various different industrial applications [11]. In this fungus, the secretory pathway is an important route of cell communication with the environment. It is responsible for secreting molecules to send distinct signals to other cells, hydrolytic enzymes to obtain nutrients and respond or adapt to changes in the microenvironment [12]. Regarding this, different organelles are responsible for the biosynthesis, assembly, folding and modifications of proteins in the secretory pathways [13]. In process of proteins synthesis, proteins are synthesized in the ribosomes and then, are transported through the rough endoplasmic reticulum (ER), and thus, the polypeptides are translocated into the lumen of this organelle [14]. Once in the ER lumen, the polypeptides may undergo various posttranslational modifications [15].

After trafficking inside the ER, the proteins are directed from ER to the Golgi complex, and this process involves packaging proteins in coat protein complex II (COPII)-coated vesicles [16, 17]. In *T. reesei*, genomic analysis has indicated the presence of the gene encoding COPII-coated vesicles for the anterograde transport from ER to Golgi complex [12, 18]. Right after proteins are delivered to the Golgi complex they undergo proteolytic processing to generate mature polypeptides [19]. Finally, in the last step of the secretory pathway, the protein needs to be transported from Golgi complex to the plasma membrane, and this process involves vesicle formation. Extracellular vesicles (EVs) are defined as a heterogeneous group of cell-derived membranous structures comprising exosomes and microvesicles, which originate from the endosomal system or which are shed from the plasma membrane, respectively [20].

During this process of vesicles formation, different regulatory proteins have an essential role. In this scenario, GTPase Arf1 has a crucial participation in the formation of the exomer, which is responsible to coat vesicles designated to the plasma membrane [21], and GTPases Sec4, Rho1, Rho3, and Cdc42 need to be activated to promote the final docking event allowing the coated-vesicles reach the plasma membrane [22, 23]. Finally, SNARE proteins such as Sec9, Snc1, Sso1, and Sso2 are equally important in vesicle and plasma membrane fusion [24]. Regarding, in *T. reesei, snc1*, *sso1*, and *sso2* have been identified in subapical areas of the hyphal plasma membrane [25].

Using bioinformatic analysis of signal sequences, Druzhinina et al. [26] suggested that the majority of the secreted proteins in *T. reesei* are hydrolases, small cysteine-rich proteins, proteases, lipases, nucleases, oxidases, and phosphatases. Furthermore, beyond the secretion of holocellulolytic enzymes, this fungus has the ability to secrete laccases, glyoxal oxidase, peroxin, peroxidase, catalase, glutathione transferase, cytochrome oxidase and cytochrome peroxidase [27]. Regarding holocellulolytic enzymes, the cellobiohydrolases (Cel7a and Cel6a) and the endoglucanases (Cel7b and Cel5a) are the most common cellulolytic enzymes identified in the secretome of this fungus [28, 29]. Though all this knowledge about cellulase secretion on *T. reesei*, there is a series of questions regarding the secretory pathway of cellulases that remain unanswered, including (1) How does cellulose trigger the induction of cellulases? (2) What are the intracellular signaling pathways? (3) What are the subcellular locations of cellulases? So, here we showed for the first time the identification and characterization of extracellular vesicles in *T. reesei* along with the proteomic content analysis of extracellular vesicles (EVs) in the presence of cellulose.

## Methods

### Fungal strain and growth conditions

The *Trichoderma reesei* strain QM6aΔ*tmus53*Δ*pyr4* [30] used in this study was obtained from the Institute of Chemical Engineering & Technical Biosciences of Vienna University of Technology, TU Vienna, Austria. The strain was maintained at 4 °C on MEX medium [malt extract 3% (w/v) and agar–agar 2% (w/v)], which was supplemented with 5 mM uridine in the case of the *pyr4* deletion strain. For all experiments, a spore suspension of QM6aΔ*tmus53*Δ*pyr4* strain containing 10^6^ cells/mL was precultured into 200 mL of Mandels–Andreotti medium [31] supplemented with glycerol 1% (w/v) for 24 h and then transferred to a 200 mL of fresh Mandels–Andreotti medium containing 1% of Avicel [32, 33]. The cultures were incubated on an orbital shaker (200 rpm) at 30 °C for 24, 48, 72, 96 or 120 h. For glucose experiments, the fungus was grown in 2% glucose for 24 h. The resulting culture supernatant was collected by filtration and used for EVs isolation. All experiments were performed in three biological replicates.

### Vesicles isolation and characterization

Extracellular vesicles were isolated as previously described for other fungi [34–37], with slight modifications. *T. reesei* fungal cells were separated from culture supernatants by centrifugation at 4000×*g* for 30 min at 4 °C, and the pellets discarded. The obtained supernatants were concentrated approximately 50-fold through polystyrene membranes using Amicon ultrafiltration system (cutoff, 100 kDa). The resulting supernatant was centrifuged at 60,000×*g* for 1 h at 4 °C. The supernatants were discarded, and the pellets washed twice with 0.1 M phosphate-buffered saline (PBS) by sequential resuspension and centrifuging them at 60,000×*g* for 1 h at 4 °C.

The size-distribution and quantification of extracellular vesicles preparations were measured by nanoparticle-tracking analysis (NTA) using a NanoSight NS300 (Malvern Instruments, Malvern, UK) equipped with fast video capture and particle-tracking software, as previously described [38]. Purified vesicles from *T. reesei* were 20× diluted into 1 mL of PBS, and each sample was then injected into a NanoSight sample cubicle. The measurements were obtained in triplicate and analyzed using NanoSight software (version 3.2.16). The data on the sizes of extracellular vesicles from *T. reesei* are expressed as the calculated mean ± SD of size distribution.

### Transmission electron microscopy (TEM)

Mycelia aliquots withdrawn from QM6aΔ*tmus53*Δ*pyr4* at 24, 48, 72, 96 and 120 h during culture in the presence of Avicel, and glucose and glycerol at 24 h were centrifuged at 6000×*g* and immediately fixed in 0.1 M sodium phosphate buffer (pH 7.4) containing 2.5% (v/v) of glutaraldehyde and 2% (w/v) of paraformaldehyde for 24 h at 4 °C. Samples were encapsulated in agar (2% w/v) and subjected to fixation (1% OsO_4_), contrasting (1% uranyl acetate), ethanol dehydration, and a two-step infiltration process with Spurr resin (Electron Microscopy Sciences) of 16 h and 3 h at room temperature (RT). Additional infiltration was provided under vacuum at RT before embedment in BEEM capsules (Electron Microscopy Sciences) and polymerization at 60 °C for 72 h. Semithin (0.5-µm) survey sections were stained with toluidine blue to identify the areas of best cell density. Ultrathin sections (60 nm) were prepared and stained again with uranyl acetate (1%) and lead citrate (2%). Transmission electron microscopy (TEM) images were obtained using a Philips CM-200 electron microscope at an acceleration voltage of 120 kV using a MegaView3 camera and iTEM 5.0 software (Olympus Soft Imaging Solutions GmbH).

### Proteomic analysis

The vesicles samples were sonicated to release protein content. Subsequently, total protein was quantified using the Bradford method and concentrated using Amicon Ultra 0.5 mL centrifugal filters (MWCO 3 kDa) (Millipore, Burlington, Massachusetts, EUA). Proteins were suspended in 250 µL 100 mM NH_4_HCO_3_ containing 8 M urea and reduced with 5 mM dithiothreitol 37 °C for 1 h. Reduced thiol groups were alkylated with 100 mM NH_4_HCO_3_ containing 8 M urea and 50 mM iodoacetamide for 1 h at RT in the dark. Protein digestion was carried out by incubating the samples with trypsin 20 ng/μL (Promega; Madison, WI, USA; 1:50; w/w) overnight at 37 °C. After quenching the reaction with trifluoroacetic acid (TFA) to a final concentration of 1% TFA, the peptide mixture was desalted, concentrated on ziptip (Millipore) [39]. Recovered peptides were dissolved in 0.1% formic acid and then subjected to nanoLC–MS/MS analysis, using Bruker Maxis Q-TOF coupled to a nanoLC (NanoAcquity Waters). The peptides were loaded onto a nanoAcquity UPLC^®^ 2G-V/MTrap 5 µm Symmetry^®^ C18 180 µm × 20 mm precolumn (Waters). The elution was made with a linear gradient of 2–85% acetonitrile in 0.1% aqueous solution of formic acid. The gradient was performed over 85 min using nanoAcquity UPLC^®^ 1.7 µm BEH130 100 µm × 100 mm (Waters) column at a flow rate of 0.3 µL/min.

Raw files were converted to .dta files for a Mascot database (Mascot software; Matrix Science; Boston, MA, USA) search. A database containing *T. reesei* protein sequences was searched using Mascot software (version 2.3) for protein identification. Moreover, acquired raw data were converted to mzXML and automatically processed by an in-house installation of Labkey Server v12, using the X!Tandem search algorithm [40]. The minimum criterion for peptide matching was performed using a Peptide Prophet [41] score greater than 0.8. Peptides that met these criteria were further grouped to protein sequences using the Protein Prophet [42] algorithm and only proteins with an error rate of 5% or less and two peptides sequences identified were considered as valid identifications. A combined list of proteins identified in all replicates (*n* = 3) and MASCOT-LabKey Server searches were condensed to remove redundant IDs such as orthologous sequences, redundant database entries, and indistinguishable isoforms based on observed peptide coverage.

### RNA isolation

RNA was extracted from lyophilized vesicles samples using the TRIzol^®^ RNA kit (Invitrogen Life Technologies, CA, USA), according to the manufacturer’s instructions. The RNA concentration was determined by spectrophotometric OD at 260/280, and the RNA integrity was verified by both the Agilent 2100 Bioanalyzer.

### Enzyme activity assays

The vesicles samples were sonicated to release protein content. Subsequently, β-glucosidase and filter paper (FPase) activities were assayed in the isolated vesicles or supernatant culture by the ability to hydrolyze pNPG (*p*-nitrophenyl-β-d-glucopyranoside) and filter paper, respectively, following published protocols [43–46]. For β-glucosidase activity, the reaction consisted of 10 μL of isolated vesicle suspension or 10 μL supernatant, 50 µL of 50 mM sodium acetate buffer and 40 µL of 5 mM of a specific substrate. The reactions were incubated at 50 °C for 15 min (β-glucosidase), followed by the addition of 100 μL of 1 M sodium carbonate [47]. The enzymatic activities were performed in a 96-well microplate, and absorbance was read at 405 nm using the xMark™ Microplate Spectrophotometer (Bio-Rad, CA, USA). Filter paper activity (FPase) was determined by an enzymatic reaction employing Whatman filter paper no. 1, 30 µL of isolated vesicle suspension or 30 µL of supernatant, and 30 µL of 100 mM citrate buffer pH 5.0 [47]. The reactions were incubated at 50 °C for 18 h. Next, 60 μL of dinitrosalicylic acid (DNS) was add to the reaction, which was then heated at 95 °C for 5 min. The FPase activity was also assayed in a 96-well microplate, and absorbance was read at 540 nm using the xMark™ Microplate Spectrophotometer (Bio-Rad, CA, USA). One enzyme unit was defined as the amount of enzyme capable of liberating 1 μmol of reducing sugar per minute [43].

## Results

### Identification and characterization of *T. reesei* extracellular vesicles

**Fig. 1:**
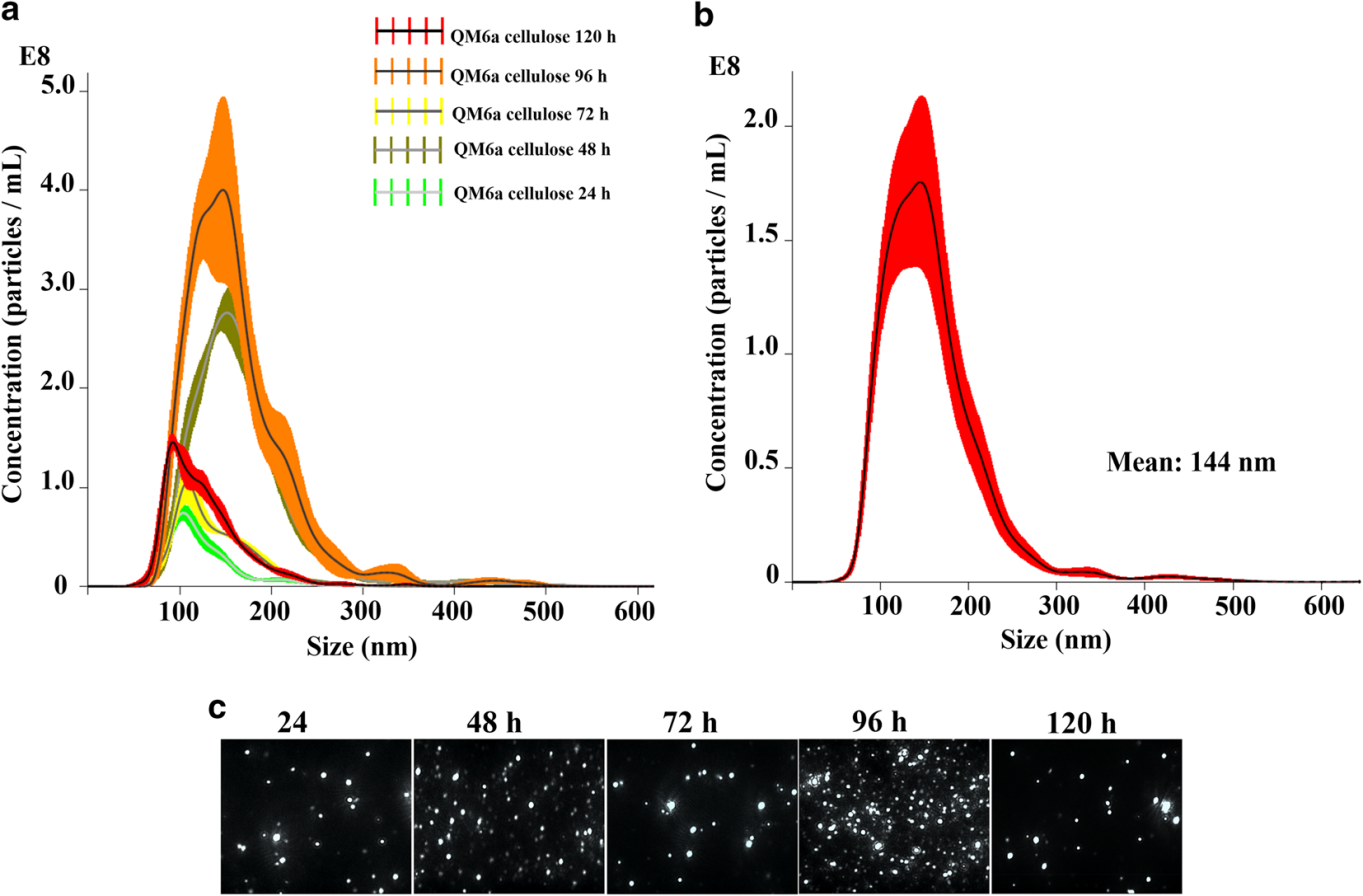
Nanoparticle-tracking analysis (NTA) of extracellular vesicles (EVs) produced by *T. reesei.* **a** Histogram showing the EV particle-size distribution (EVs × 10^8^/mL vs size in nanometers) from the cellulose-supernatant culture at 24, 48, 72, 96 and 120 h post-induction in the presence of cellulose. **b** Histogram showing the calculated mean ± SD of size distribution by NTA analysis of purified *T. reesei* EVs. **c** Screenshots from video recorded using NanoSight NS300, showing the distribution of EVs from the cellulose-supernatant culture at 24, 48, 72, 96 and 120 h. These results are based on three replicates of three independent experiments

This work shows the first characterization of extracellular vesicles (EVs) of *T. reesei* (Fig. 1). Our results showed that *T. reesei* was able to produce a significant number of extracellular vesicles from 24 h of induction in the presence cellulose exhibiting the highest level of production at 96 h (Fig. 1a, c). The majority of the EVs population displayed diameter size ranging from 100 to 200 nm, with a mean size of 144 nm (Fig. 1b and Additional file 1: Fig. S1). Figure 1a–c shows a representative view of the different vesicles sizes and location obtained by supernatant purification. The mean concentration of particles in the supernatants from 24, 48, 72, 96 and 120 h were 4.61 × 10^9^, 2.83 × 10^10^, 7.89 × 10^9^, 4.11 × 10^10^ and 1.09 × 10^10^ particles/mL, respectively. As expected, no particles were found in the medium alone (negative control; data not shown). Also, to confirm that the vesicles originated from live cells and not from membranes released from dead cells, we analyzed a control preparation from each time of culture supernatants of *T. reesei* mycelia that had been killed by heating at 70 °C for 24 h and then inoculated in minimal medium and cultivated in parallel under the same conditions as those used for the live cells. Our NTA results demonstrated that no nanoparticles were generated from the heat-killed *T. reesei* mycelia (data not shown).

Interestingly, our analysis demonstrated the growth of *T. reesei* in the presence of cellulose generate a higher number of EVs than compared to the repressive or neutral carbon sources glucose or glycerol, respectively (Additional file 1: Fig. S3c). As shown in this figure, in the presence of glucose and glycerol we have a few numbers of EVs. The NTA analysis showed that the EVs purified from glycerol are smaller than glucose and cellulose culture, exhibiting a size of 108 nm, 153 nm, and 144 nm, respectively (Additional file 1: Fig. S3a and b). These results suggest that there is an unknown mechanism involving EVs production in *T. reesei* in the presence of cellulose.

**Fig. 2:**
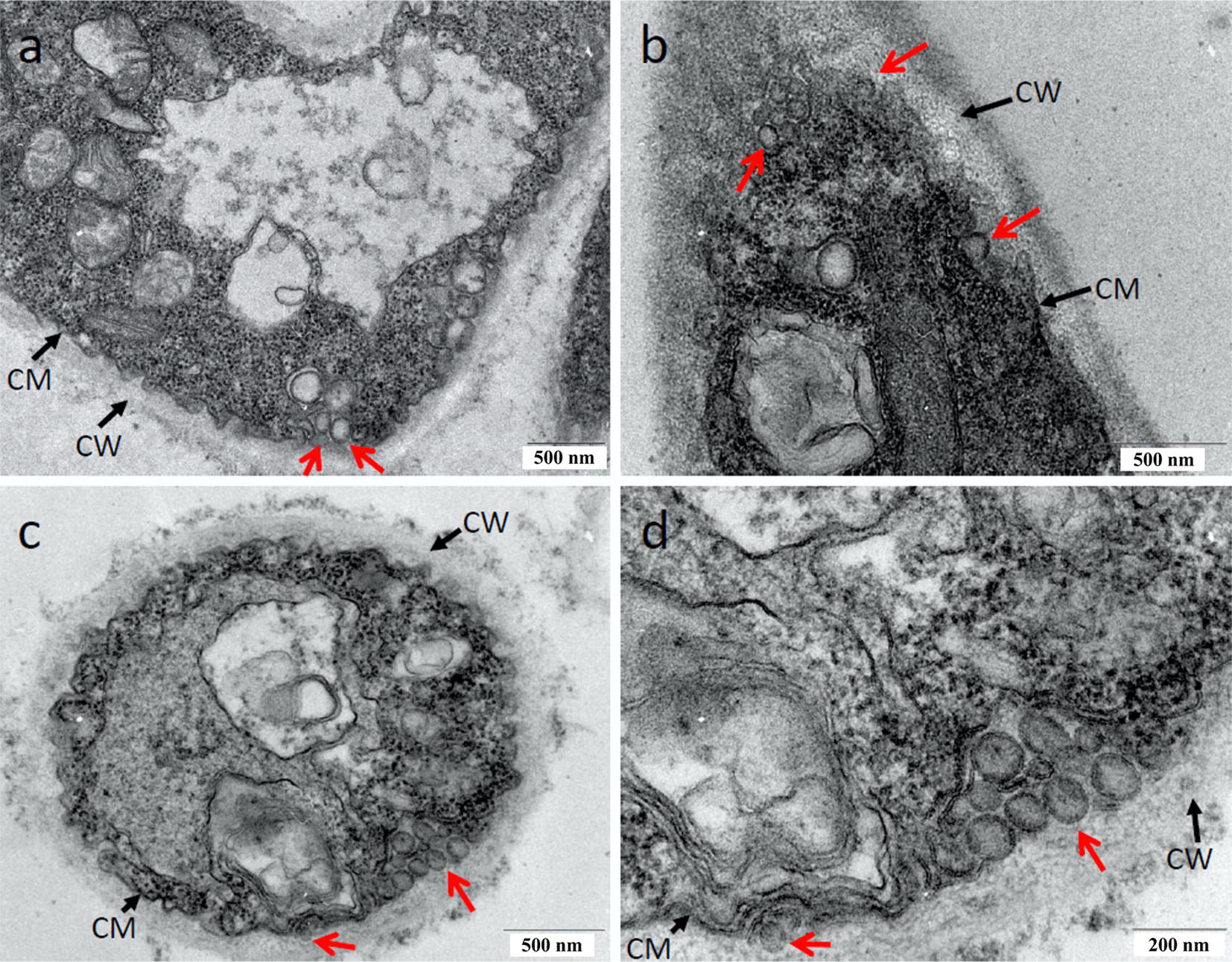
TEM analyses of vesicles in *Trichoderma reesei* mycelium cells. **a**–**d** The occurrence of vesicles in association with the cytoplasmic membrane and cell wall is evident after growth for 48 h in the presence of cellulose. Black arrows indicate the cell wall (CW) and cell membrane (CM). Red arrows indicate the *T. reesei* vesicles. Bars, 500 nm (**a**–**c**) and 200 nm (**d**)

Transmission electron microscopy analysis of vesicles from mycelium revealed a large amount of EVs in samples isolated from *T. reesei* cellulose culture (Fig. 2). The vesicles seem to be mainly associated with the cytoplasmic membrane (CM) (Fig. 2a, c) and some of them were visualized in association with the cell wall (CW) (Fig. 2b, d). Similarly, we also able to identify vesicles from mycelium cultivated in the presence of glycerol and glucose (Additional file 1: Fig. S4). However, our results showed that mycelia isolated from these conditions have a small number of EVs when compared to cellulose (Fig. 2). These results suggest that vesicles of *T. reesei* are produced in the presence of cellulose, glucose, and glycerol and are secreted via the canonical secretory pathway involving passage through the cell wall, in turn, releasing extracellular proteins to the extracellular space, thus providing additional evidence for the existence of an extracellular vesicular transport mechanism.

### Proteomic analysis

**Fig. 3:**
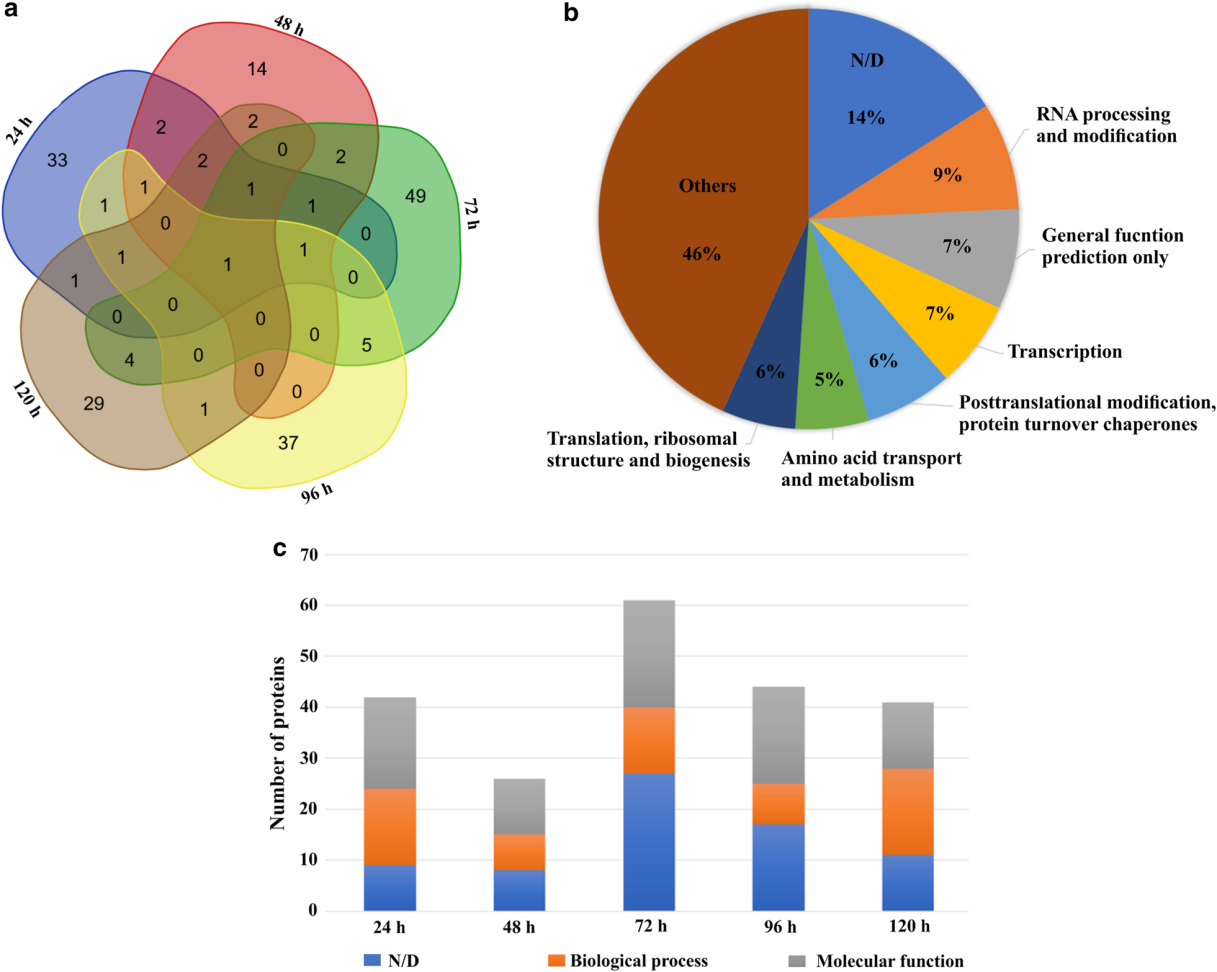
Proteomic analysis of *T. reesei* EVs. **a** Expression pattern of the proteins identified in *T. reesei* EVs at 24, 48, 72, 96 and 120 h in the presence of cellulose. Venn diagram clustering was designed using Bioinformatics & Evolutionary Genomics tools (http://bioinformatics.psb.ugent.be/webtools/Venn/). **b** KOG term of 188 proteins identified in *T. reesei* EVs. **c** Molecular function and biological process of proteins identified at 24, 48, 72, 96 and 120 h in the presence of cellulose. The proteins are classified in terms of Gene Ontology (GO) being molecular function related to proteins features, such as binding or catalysis, and finally, biological process, classified the proteins according to the processes that it involved. These results are based on three replicates of three independent experiments

In order to analyze the effects of cellulases secretion in response to cellulose, we identified the protein content of the EVs. Our report contains the first EVs proteomic analysis of *T. reesei* during growth in the presence of an inductive carbon source. Accordingly, EVs were isolated from cell-free culture supernatants from *T. reesei* and analyzed by LC–MS/MS. A total of 188 proteins were identified in a time-course experiment (24 h to 120 h) of cultivation in the presence of cellulose (Additional file 2). Among these proteins, 33 of them were identified exclusively in vesicles originating from cell-free culture supernatant induction at 24 h, 14 found exclusively at 48 h, 49 shown exclusively at 72 h, 37 found exclusively at 96 h and 29 identified exclusively at 120 h in the presence of cellulose (Fig. 3a). These results strongly suggest dynamics in vesicle content production during the cellulose utilization by the fungus. Consistently, a small number of proteins were commonly identified among the conditions examined and only one protein, encoding an RNA-binding protein involved with rRNA biogenesis (ID 78062) was identified in all time points (Fig. 3a). Furthermore, our data showed that 12 proteins with chaperones-related activity, which are involved in folding protein, were identified inside the *T. reesei* EVs (Table 1). These results suggest that protein folding, stability, and modifications to reach native structure could be an active process inside *T. reesei* EVs.

**Table 1:** Chaperone-like proteins identified inside *T. reesei* extracellular vesicles

Protein ID	Description	GO	KOG	Identification time
119924	Hsp70 nucleotide exchange factor FES1, putative	#N/D	Posttranslational modification, protein turnover, chaperones	24 h
119731	Heat shock protein hsp60 mitochondrial precursor protein	Cellular protein metabolism	Posttranslational modification, protein turnover, chaperones	24 h, 48 h, 72 h and 120 h
66183	Peptidase_Serine carboxypeptidase	Proteolysis and peptidolysis	Posttranslational modification, protein turnover, chaperones	24 h
73678	Calnexin, high identity with *A. niger* clxA	Calcium ion binding	Posttranslational modification, protein turnover, chaperones	24 h
36822	Unknown protein	Catalytic activity	Posttranslational modification, protein turnover, chaperones	24 h
48001	Unknown protein with DnaJ domain	Unfolded protein binding	Posttranslational modification, protein turnover, chaperones	48 h
107011	Unknown protein	Unfolded protein binding	Posttranslational modification, protein turnover, chaperones	72 h
104390	Glutathione *S*-transferase	Glutathione transferase activity	Posttranslational modification, protein turnover, chaperones	72 h
124282	Unknown protein, SET and MYND domain	#N/D	Posttranslational modification, protein turnover, chaperones	96 h
21444	Unknown protein	Hydrogen-transporting ATPase activity, rotational mechanism	Posttranslational modification, protein turnover, chaperones	120 h
21461	ATP-dependent protease La, putative	ATP binding	Posttranslational modification, protein turnover, chaperones	120 h
123922	Peptidyl-prolyl isomerase	Protein folding	Posttranslational modification, protein turnover, chaperones	72 h and 96 h

The vesicular proteins were categorized by the biological process using KOG annotation and was demonstrated that they are involved (Fig. 3b) with six principal groups, including, 9% RNA processing-related proteins, 7% with general functions, 7% transcription-related proteins, 6% of proteins associated to posttranslational modification, 5% involved with amino acid transport and metabolism and 6% translation-related proteins. Regarding the remaining proteins, including CAZymes, 46% were related to other functions and 14% are still uncharacterized. Again, our time-course categorization by molecular function and biological process showed that the functional composition of *T. reesei* vesicles content is very diverse (Fig. 3c). These results suggest that the protein content of the vesicles is highly variable, and possibly correlated with different growth stages of the fungus during cultivation in presence of cellulose.

### CAZy-related proteins are enriched inside *T. reesei* EVs

Interestingly, our proteomic analysis showed that there are some CAZy-related proteins inside de *T. reesei* EVs after growth in the presence of cellulose (Table 2). In this respect, we identified nine CAZYmes inside the EVs including, a glycoside hydrolase (GH) GH64 endo-1,3-β-glucanase, a GH36 α-galactosidase (AGL2), a GH55 candidate exo-1,3-β-glucanase, two glycosyltransferases (β-1,6,*N*-acetylglucosaminyltransferase), a GH72 (candidate membrane-bound β-1,3-glucanosyltransglycosylase), and three proteins related to chitinolytic enzyme machinery of fungi (GH18 chitinase Chi18-5, a GH20 exochitinase nag1—possibly involved with mycoparasitism and GT2 chitin synthase chs3—involved with chitin biosynthesis). Finally, we demonstrated that the cellobiohydrolase Cel7a belonging to GH7 family, the dominant enzyme of the *T. reesei* cellulolytic complex was also identified in the EVs cellulose proteome.

**Table 2:** CAZy-related proteins identified inside *T. reesei* extracellular vesicles

Protein ID	Description	GO	KOG	Identification time
124175	GH64 endo-1,3-β-glucanase	#N/D	#N/D	72 h and 120 h
124016	GH36 α-galactosidase AGL2	Carbohydrate metabolism	#N/D	96 h and 120 h
121746	Glycoside hydrolase family 55 (candidate exo-1,3-b-glucanase)	Carbohydrate metabolism	#N/D	72 h and 120 h
123649	β-1,6-*N*-Acetylglucosaminyltransferase	Carbohydrate transport and metabolism	Carbohydrate transport and metabolism	72 h and 96 h
123538	Candidate membrane-bound β-1,3-glucanosyltransglycosylase (GH72 β-1 3-glucanosyltransferase)	#N/D	#N/D	48 h
80833	GH18 chitinase Chi18-5	Hydrolase activity	Carbohydrate transport and metabolism	72 h
21725	GH20 exochitinase (nag1)	Carbohydrate metabolism	Carbohydrate transport and metabolism	120 h
123989	Glycoside hydrolase family 7 (cel7a)	Carbohydrate metabolism	Function unknown	48 h and 120 h
112271	GT2 chitin synthase chs3	Chitin biosynthesis	Cell wall/membrane/envelope biogenesis	72 h

**Fig. 4:**
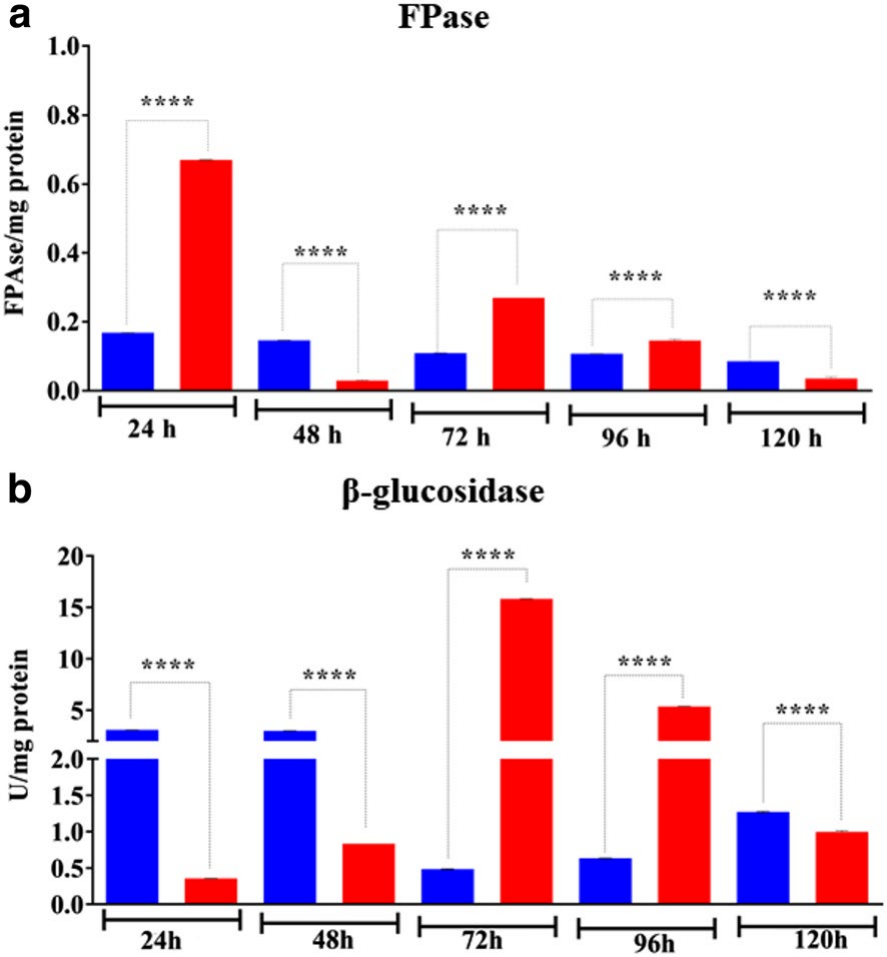
Cellulolytic activities from culture supernatant and purified *T. reesei* EVs after induction in the presence of cellulose. **a** Filter paper activity (FPase) and **b** β-glucosidase activity from culture supernatant (blue bars) and purified *T. reesei* EVs (red bars) grown at 24, 48, 72, 96 and 120 h in the presence of cellulose. ****Significantly different (P < 0.001). These results are based on three replicates of three independent experiments and are expressed as mean ± standard deviation

In addition, enzymatic assay revealed that the specific filter paper activity (FPase) was higher in purified EVs from 24, 72, and 96 h of cultivation in presence of cellulose when compared to culture supernatant at the same time (Fig. 4a). The lowest FPase activity was detected inside vesicles from 48 to 120 h of cultivation in the presence of cellulose. Curiously, although our proteomic analysis was not able to identify β-glucosidase inside cellulose-purified EVs, the specific β-glucosidase activity was higher in purified EVs from 72 to 96 h of cultivation when compared to culture supernatant at the same time (Fig. 4b). In the same assay, cellulose-purified EVs at 24 h, 48 h, and 120 h presented significantly lower β-glucosidase activity than the supernatant culture. Our enzymatic assay revealed that EVs isolated from glucose and glycerol showed almost undetectable level of cellulase activity (Additional file 1: Fig. S5). These results suggest that there is an intriguing mechanism of β-glucosidase and total cellulase enrichment inside the vesicles in the presence of cellulose.

Concomitantly, our RNA analysis revealed that there is a slight enrichment of RNA inside vesicles from 96- and 120 h of induction by cellulose. This content consisted mostly of EVs RNA molecules less than 200 nucleotides (Additional file 1: Fig. S2). This result suggests that *T. reesei* is able to release smalls RNAs from extracellular vesicles and these RNA-containing vesicles may be important to regulate various biological processes in this fungus.

## Discussion

Previous studies with other organisms such as *Saccharomyces cerevisiae* [48], *Cryptococcus neoformans* [49] and *Histoplasma capsulatum* [50] revealed that fungal extracellular vesicles carry proteins with highly diverse functions. Similarly, our data revealed that the extracellular vesicles of *T. reesei* have a wide range of proteins (Additional file 2). The same way, our proteomic analyses also pointed out important findings of vesicle formation and transport in *T. reesei* in response to cellulose presence. The coat protein complex II (COPII) generates transport vesicles that mediate protein transport from the endoplasmic reticulum (ER) [17]. After synthesis, most of the secretory proteins within the endoplasmic reticulum are transported to the Golgi via COPII-coated vesicles and, the formation of COPII-coated vesicles is regulated by small GTPases [51, 52]. Then, assembly of the COPII coat is initiated through activation of the small Ras-like GTPases by ER-bound transmembrane guanine-nucleotide exchange factor (GEF), promoting the recruitment of Sec23/Sec24, an inner-coat complex component, by activated-small GTPases to form the pre-budding complex with cargo proteins [53, 54].

Our results demonstrated that some components involved with intracellular trafficking, secretion, and vesicular transport such as vesicle coat complex COPII-Sec24 and small GTPases, belonging to COPII-GTPAse-Sec24 secretion pathway were identified at EVs proteome in *T. reesei* in presence of cellulose. Similarly, we demonstrated that induction promoted by cellulose led to the production of EVs enriched with specific small GTPases such as IDs 80898 and 77031, which are involved in polar growth, exocytosis, endocytosis and secretory-vesicle fusion to the plasma membrane [55–57]. In addition, a vesicle-associated membrane protein (VAMP/Synaptobrevin) involved with trafficking proteins were equally identified in our data. This protein is a crucial component of *trans*-Golgi network (TGN)-endosomal system for vesicular transport, being responsible to the export, sorting, and recycling of numerous soluble and membrane-associated lysosomal and secretory pathway proteins [58, 59]. Interestingly, it has been shown that fungal cell wall plays an important role in the secretion process since many of the proteins secreted are structurally associated with the plasma membrane and the cell wall [60].

Furthermore, a number of cellular processes such as secretion involve the transport of intracellular membrane vesicles that may be attended by the actin cytoskeleton remodeling [55]. Here, we identified several proteins including actin and tubulin structural molecules associated with microtubule-based movement inside *T. reesei* EVs supporting the evidence for a vesicle trafficking under the presence of cellulose from 24 to 120 h of culture. These results provide evidence to a canonical mechanism of protein secretion in this fungus, involving the formation and recruitment of new proteins for constructing secretory pathways in the presence of cellulose. *T. reesei* EVs were also enriched with proteins involved with posttranslational modification, protein turnover, and chaperones. Among them, we identified two heat shock proteins (Hsp70 and Hsp60), a carboxypeptidase, a peptidyl-prolyl isomerase, a molecular chaperone protein with a DnaJ domain and an ER-chaperone calnexin located in the membrane of the endoplasmic reticulum to ensure the proper folding of glycoproteins [61]. These findings suggest that there is an appropriated folding process involving the formation of disulphide bond to develop the tertiary and quaternary structures of the protein to provide a proper protein secretion process in response to an inductive carbon source. Moreover, our results identified for the first time the presence of Hsp proteins inside *T. reesei* EVs. Hsp proteins have been suggested to be exported by an interesting mechanism mediated by the insertion of the protein into the membrane of export vesicles [62, 63]. O’Neill and Quah [64] have suggested that extracellular Hsp are likely to act as indicators of the stress conditions, priming other cells, particularly of the immune system, to avoid the propagation of the insult. Additionally, so extracellular Hsp, for instance, Hsp70, is associated with export vesicles, displaying a robust activation of macrophages [64]. Together, our results suggest that Hsp proteins associated with *T. reesei* EVs might be an additional mechanism of *T. reesei* to environmental sensing by the fungus, and future studies are required to better understand their importance in EVs and possible involved mechanisms.

About the holocellulolytic biomass degradation system of *T. reesei*, it has been shown that is one of the most studied within the filamentous fungi. This fungus has the ability to produce a high cellulase content due to the efficient capability to sense and transport nutrient for rapid induction and secretion of cellulases [32, 65–67]. However, there is a series of questions regarding the secretory pathway of cellulases that remain unanswered. Since cellulases are secreted enzymes, the control of their biosynthesis may require various steps of the secretory pathway [68]. In *T. reesei*, studies have shown that cellulases are resident in the endoplasmic reticulum-derived vesicles attached to the outside surface of the membrane [69]. First of all, the cellulase expression and secretion requires an induction that might include the formation of new proteins for constructing secretory pathways [70]. *T. reesei* secrets large amounts of extracellular cellulolytic enzymes [71, 72] which are regulated in a coordinated manner that depends on the availability of the carbon sources [32, 66, 73, 74]. However, there is an open discussion about the nature of such an inducer molecule, since cellulose is unlikely to directly trigger the cellulase induction because of its insolubility [75]. Here, we showed that in the presence of cellulose *T. reesei* is able to produce a high concentration of EVs when compared to glucose and glycerol. In the presence of glucose, a readily available carbon source, the fungus can activate the mechanism of catabolite carbon repression (CCR) and inhibit the production of cellulolytic complex enzymes to avoid unnecessary energy expenditure [6, 76]. This way, our results suggest that growth in the presence of cellulose can promote an increase in EVs production and these particles might be enriched with cellulases through an unclear mechanism yet. Our results are according to those observed in *Fibrobacter succinogenes,* a cellulolytic bacterium, in which it was showed that cellulose induces vesicles formation [77]. This way, our results suggest that *Fibrobacter* and *Trichoderma* might share some physiological similarities in EVs formation. However, even with these similarities, the mechanisms involved with EVs formation in both organisms might be different in various aspects. So, further experiments are necessary to fully understanding of the factors controlling EVs formation in *T. reesei.* At this moment, we were able to show that there is a presence of cellulase inside *T. reesei* EVs, and this could be involved in cellulose degradation as supported by the presence of CAZymes inside EVs as demonstrated by our proteomic analysis.

In this context, reports have suggested that there are different mechanisms underlying cellulase secretion, one specific secretory pathway independent of cellulose, another induced by cellulose, and a third one that occurs separately of the carbon source [78]. So, how would cellulose trigger the induction of cellulases? Different studies investigating this aspect postulated the induction function of a low molecular weight and soluble compound derived from cellulose [79, 80]. A hypothesis is that *T. reesei* has low levels of constitutive cellulase promoting the cellulose breakdown when it becomes available [81]. Gong and Tsao [82] and Stutzenberger [83] proposed a model for the first steps of the cellulose degradation process through the action of cellulases. These authors suggested the conidia surface contains cellobiohydrolases that degrade the cellulose into cello-oligosaccharides, which in turn may be either hydrolyzed until glucose for immediate consumption or transglycosylated to sophorose, by the action of a constitutive β-glucosidase. The sophorose obtained from the cello-oligosaccharides processing then acts as an inducer for de novo production and secretion of cellulases, starting a cycle where glucose and sophorose are produced until complete cellulose degradation. It was also demonstrated that the cellobiohydrolases Cel7a and Cel6a, and the endoglucanase Cel5a are constitutive of the conidia surface and, along with the β-glucosidase, these enzymes are crucial for the initiation of cellulases induction for cellulose breakdown [84, 85]. Similarly, the existence of a constitutive β-glucosidase on the plasma membrane has also been suggested [86, 87]. Foreman et al. [88] also suggested that CEL5B contains the consensus sequence for membrane-anchoring via a glycosylphosphatidylinositol residue which make it an interesting candidate for generating the inducer of cellulase formation. Similarly, the acetyl xylan esterase Axe2, which is also predicted to contain a glycosylphosphatidylinositol anchor, may be involved in the primary induction of some hemicellulases [88]. Therefore, we argue that *T. reesei* secrets vesicles containing cellulases that in turn releases cellobiose and glucose for cellulase induction and production, as well as glucose for fungal growth. However, as discussed above the mechanisms of induction of cellulase expression are complex and some aspects are unclear yet. Probably, there is crosstalk between different molecular mechanisms involved in cellulase formation. This way, our results brings light into how this process can trigger in *T. reesei*. Nevertheless, there is a still undefined role in regulated mechanisms of cellulase induction and secretion in this fungus, and future studies are required to better understand their importance in EVs and possible involved mechanisms. Furthermore, as an additional mechanism, *T. reesei* can secrete heat-shock proteins since these proteins were identified in EVs indicating novel strategies of environmental sensing by the fungus.

Burnet et al. [89] showed that *Fibrobacter succinogenes*, an important degrader of lignocellulosic plant material in the herbivore gut is able to produce extracellular vesicles. Forsberg et al. [90] suggested the use of these vesicles in cellulose degradation. According to this author, vesicles containing cellulases are released from the outer membrane via bleb formation. They posited that these vesicles could be released into the area between the cellulose and cell. Additionally, they demonstrated vesicles adhering to cellulose and vesicles either attached to the outer membrane or in the extracellular medium, but only in the stationary cultures. Also, is suggested that the use of vesicles during stationary phase may act as a delivery mechanism for a concentrated cocktail of cellulases to actively degrade cellulose at the site of attachment [90]. This way, cellulose is degraded and, the resulting cellodextrins would be transported into the cell for further breakdown. When attached cells reach stationary phase, the outer membrane blebs of and forms vesicles in order to continue cellulose degradation in a nutritionally limited environment [91]. These findings are consistent with a previous report showing that the presence of cellulose induces vesicles formation [77].

Likewise, Arntzen et al. [92] showed that OMVs (Outer Membrane Vesicles) produced by *F. succinogenes* are equipped with a diverse suite of enzymes able to depolymerize most common plant polysaccharides, including cellulose. Moreover, OMVs might assist the metabolism of the host cell by deconstructing non-essential polysaccharides that restrict access to the host’s target carbon source [92]. Finally, in *F. succinogenes,* cellulose binding proteins are arranged in novel putative complexes in OMVs, suggesting that this bacterium degrades biomass using means that differ fundamentally from well-known degradative machinery in nature. As proposed by *F*. *succinogenes, T. reesei* can use EVs to secrete cellulases to make the process of cellulose degradation more efficiently. Moreover, the production of EVs enriched with CAZymes might be in response to an inductive carbon source as proposed by [77], contributing to a favorable process of the economy of energy.

Recently, it was demonstrated that plants packages small RNAs in extracellular vesicles directed to fungal pathogens to silence virulence genes [93], showing that plant extracellular vesicles (mainly exosomes) have a crucial role in cross-kingdom sRNA trafficking between *Arabidopsis* and the fungal pathogen *Botrytis cinerea*. Likewise, the human pathogens *C. neoformans*, *Paracoccidioides brasiliensis,* and *Candida albicans*, and the model yeast *S. cerevisiae* can accumulate small RNAs inside extracellular vesicles [37]. Also, in *H. capsulatum,* EVs carry RNA-binding proteins, as well as mRNAs and non-coding RNAs [94]. The process of RNA export mediated by EVs have been discussed as a universal mechanism for inter-kingdom and intra-kingdom communication [95], and it is reasonable to suggest that RNAs transported by fungal EVs might play important role in cell communication [96]. Since EVs were reported involved in reverted avirulent phenotype by mechanisms vesicular RNA dependent in *Cryptococcus gattii* [97], it is also reasonable to suggest that enrichment of small RNAs in the EVs 96- and 120 h in *T. reesei*, might have a still undefined role in regulated mechanisms of gene expression in this fungus, and future studies are required to better understand their importance in EVs and possible involved mechanisms.

The presence of numerous secretion pathways in filamentous fungi might provide information about their high secretory capacity. Nevertheless, the molecular machinery for protein secretion in filamentous fungi remains mostly unknown [98]. There are many unclear issues regarding fungal EVs biosynthesis and unloading. Thus, to understand the biological functions of fungal EVs should reveal important roles of EVs in several biological processes [96]. Galectin-3, a mammalian β-galactoside-binding protein, has been reported playing disruption and internalization of EVs produced from *C. neoformans* [35] and *P. brasiliensis* [99] by macrophages. However, additional studies are needed to discern how EVs deliver their cargo, as well as to identify new molecules playing a lytic effect on fungal EVs. Briefly, our data showed the first characterization and proteomic analysis of *T. reesei* EVs in the presence of cellulose. Here, we present some new evidence supporting that the secretory pathway from *T. reesei* has the basic machinery for vesicle trafficking, similar to other filamentous fungi and eukaryotes. In initial signaling in presence of cellulose, *T. reesei* secretes a great number of vesicles enriched with cellulases and heat shock proteins (Hsps) to breakdown the cellulose polymer and, for carbon source recognition and environmental sensing, respectively. Then, cellobiose released after cellulose degradation can be both cleaved to glucose or transglycosylated to form sophorose. In turn, glucose may be used for fungal growth while cellobiose and sophorose may induce the cellulase expression by regulation of the main transcription factors involved with the control of cellulase expression. Finally, the synthesized cellulases might be packaging in vesicles and released to an environment to ensure cellulose breakdown. The data open new approach in order to generate mutants to increase biomass degradation.

## Conclusions

Currently, the filamentous fungi *T. reesei* have been established as the most important holocellulase producer used by biotechnology industries. Thus, genomics, transcriptomics, proteomics, and secretomics analyses have been employed as excellent approaches to the better understanding of mechanisms involved with cellulase gene expression and secretion in this fungus. This way, knowledge about the mechanisms involved in the recognition of environmental signals is extremely important in fungal biology comprehension. In conclusion, our study brings new insights about the cellulase secretion in *T. reesei* and suggests that there are additional secretory pathways involved in cellulase secretion in this fungus since a few numbers of cellulases were found associated with cellulose-purified EVs.

## Additional files

**Additional file 1: Fig. S1.** Characterization of *Trichoderma reesei* EVs. Mean size distribution by Nanoparticle Tracking Analysis (NTA) of purified *T. reesei* EVs from the cellulose-supernatant culture at 24, 48, 72, 96 and 120 h. These results are based on three replicates of three independent experiments. **Fig. S2.** Bioanalyzer profile of the RNA content of EVs from the *T. reesei* fungus grown at 24, 72, 96 and 120 h in the presence of cellulose. **Fig. S3.** Characterization of *Trichoderma reesei* EVs. Mean size distribution by Nanoparticle Tracking Analysis (NTA) of purified *T. reesei* EVs from glycerol **(a)**, glucose **(b),** and both conditions **(c)** at 24 h. These results are based on three replicates of three independent experiments. **Fig. S4** TEM analyses of vesicles in *Trichoderma reesei* mycelium cells. The occurrence of vesicles in association with the cytoplasmic membrane and cell wall is evident after growth for 24 h in the presence of glucose and glycerol. Black arrows indicate the CW (cell wall) and CM (cell membrane). Red arrows indicate the *T. reesei* vesicles. Bars, 500 nm and 200 nm. **Fig. S5** Cellulolytic activities from purified *T. reesei* EVs after growth in the presence of cellulose, glycerol and glucose. **(a)** Filter paper activity (FPase) and **(b)** β-glucosidase activity from purified *T. reesei* EVs grown at 24 h in the presence of respective carbon source. **** = Significantly different (P<0.001). These results are based on three replicates of three independent experiments and are expressed as mean ± standard deviation.

**Additional file 2.** List of vesicular proteins identified in *T. reesei* EVs in the presence of cellulose.

## Data Availability

All data supporting our findings can be found in Additional files 1 and 2, which have been provided as additional materials.

## Abbreviations

EVs: extracellular vesicles
ER: endoplasmic reticulum
MEX: malt extract
NTA: nanoparticle-tracking analysis
TEM: transmission electron microscopy
pNPG: *p*-nitrophenyl-β-d-glucopyranoside
FPase: filter paper activity
CM: cytoplasmic membrane
CW: cell wall
KOG: EuKaryotic Orthologous Groups
CAZy: Carbohydrate-Active enZYmes
