# Supplementary material for: Extracellular vesicles carry cellulases in the industrial fungus Trichoderma reesei
Source: Biotechnol Biofuels. 2019 Jun 15;12:146. doi: 10.1186/s13068-019-1487-7 (PMC6570945; doi:10.1186/s13068-019-1487-7)
Supplement: Supplementary file 1 — Additional file 1: Fig. S1. Characterization of Trichoderma reesei EVs. Mean size distribution by Nanoparticle Tracking Analysis (NTA) of purified T. reesei EVs from the cellulose-supernatant culture at 24, 48, 72, 96 and 120 h. These results are based on three replicates of three independent experiments. Fig. S2. Bioanalyzer profile of the RNA content of EVs from the T. reesei fungus grown at 24, 72, 96 and 120 h in the presence of cellulose. Fig. S3. Characterization of Trichoderma reesei EVs. Mean size distribution by Nanoparticle Tracking Analysis (NTA) of purified T. reesei EVs from glycerol (a), glucose (b), and both conditions (c) at 24 h. These results are based on three replicates of three independent experiments. Fig. S4 TEM analyses of vesicles in Trichoderma reesei mycelium cells. The occurrence of vesicles in association with the cytoplasmic membrane and cell wall is evident after growth for 24 h in the presence of glucose and glycerol. Black arrows indicate the CW (cell wall) and CM (cell membrane). Red arrows indicate the T. reesei vesicles. Bars, 500 nm and 200 nm. Fig. S5 Cellulolytic activities from purified T. reesei EVs after growth in the presence of cellulose, glycerol and glucose. (a) Filter paper activity (FPase) and (b) β-glucosidase activity from purified T. reesei EVs grown at 24 h in the presence of respective carbon source. **** = Significantly different (P<0.001). These results are based on three replicates of three independent experiments and are expressed as mean ± standard deviation. [file 13068_2019_1487_MOESM1_ESM.docx]

**Extracellular vesicles carry cellulases in the industrial fungus *Trichoderma reesei***

Renato Graciano de Paula^1^, Amanda Cristina Campos Antoniêto^1^, Karoline Maria Vieira Nogueira^1^, Liliane Fraga Costa Ribeiro^1^, Marina Campos Rocha^2^, Iran Malavazi^2^, Fausto Almeida^1^ and Roberto Nascimento Silva^1^*

^1^Departamento de Bioquímica e Imunologia, Faculdade de Medicina de Ribeirão Preto (FMRP), Universidade de São Paulo, Ribeirão Preto, São Paulo, Brazil, renatogpaula@usp.br, amandaantonieto@yahoo.com.br, karolmvnogueira@hotmail.com, liliane@umbc.edu and fbralmeida@gmail.com, ^2^Departamento de Genética e Evolução, Centro de Ciências Biológicas e da Saúde, Universidade Federal de São Carlos, São Paulo, Brazil, marinacamposrocha@gmail.com and imalavazi@ufscar.br

*Correspondence to: Roberto Nascimento Silva

Department of Biochemistry and Immunology

Ribeirao Preto Medical School, University of Sao Paulo

Ribeirao Preto 14049-900, SP, Brazil, Tel.: +55 16 3602 3112,

Fax: +55 16 3602-0219, E-mail: [rsilva@fmrp.usp.br](mailto:rsilva@fmrp.usp.br).


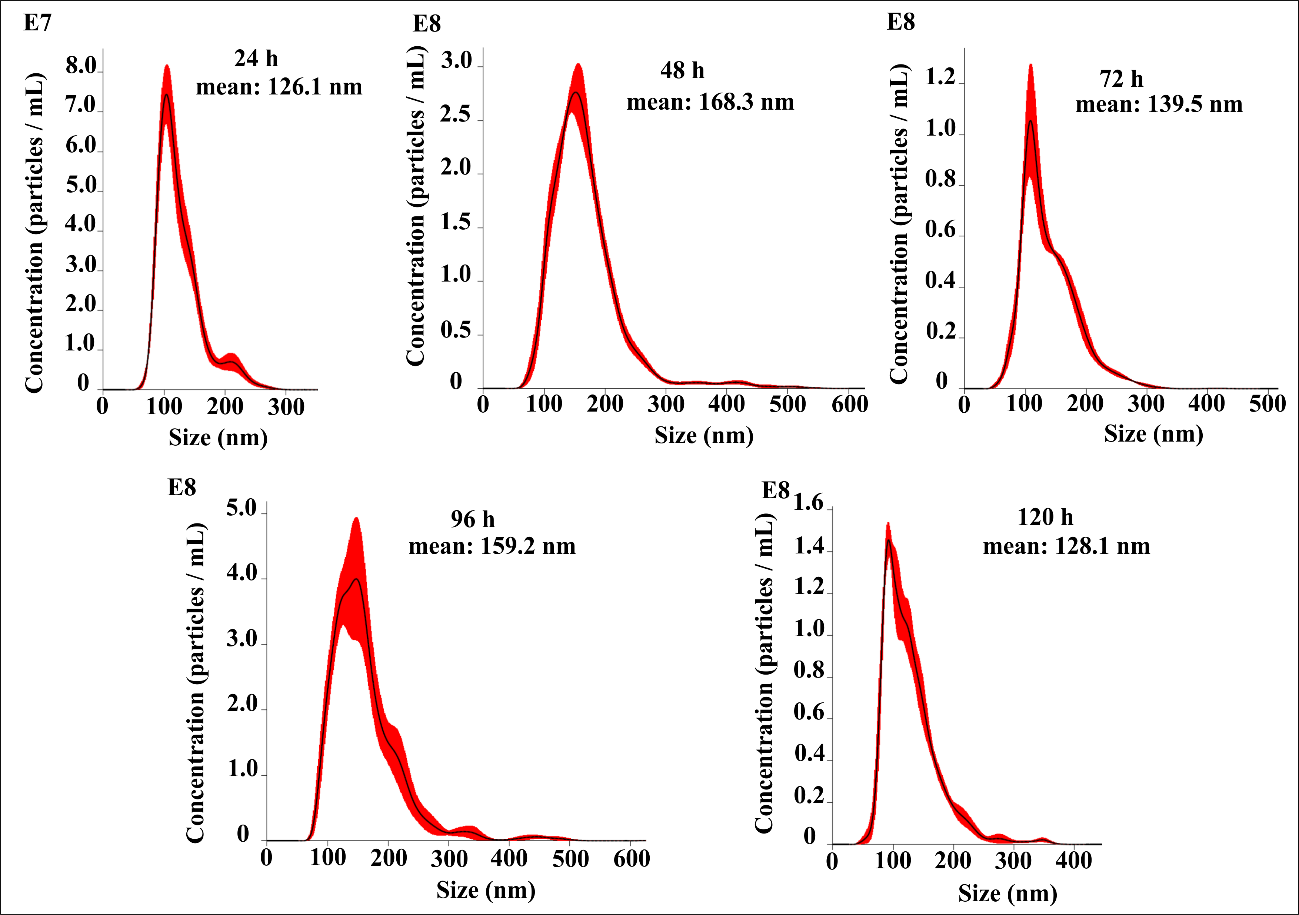


**Fig. S1** Characterization of *Trichoderma reesei* EVs. Mean size distribution by Nanoparticle Tracking Analysis (NTA) of purified *T. reesei* EVs from cellulose-supernatant culture at 24, 48, 72, 96 and 120 h. These results are based on three replicates of three independent experiments.


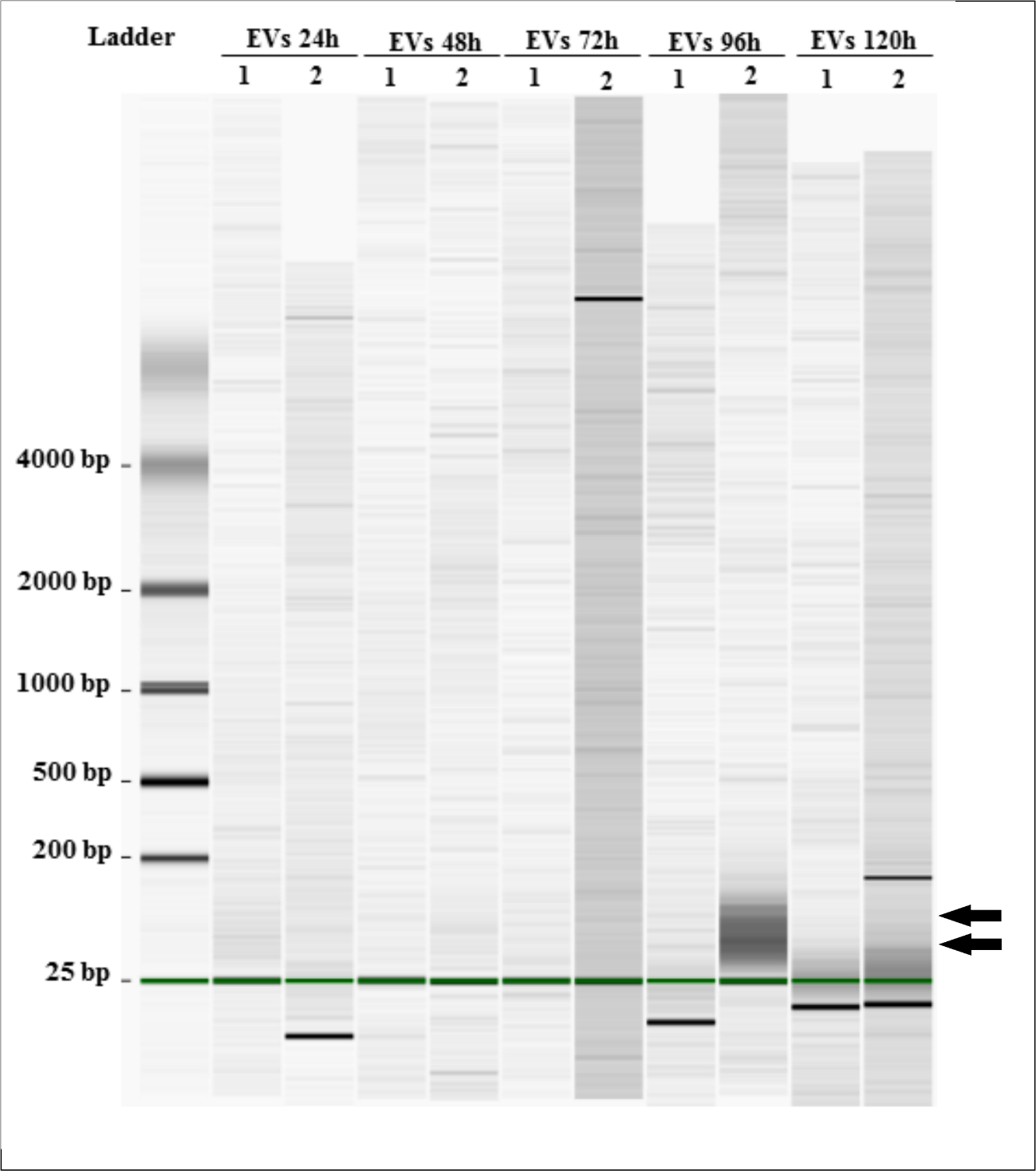


**Fig. S2** Bioanalyzer profile of the RNA content of EVs from the *T. reesei* fungus grown at 24, 72, 96 and 120 h in the presence of cellulose.


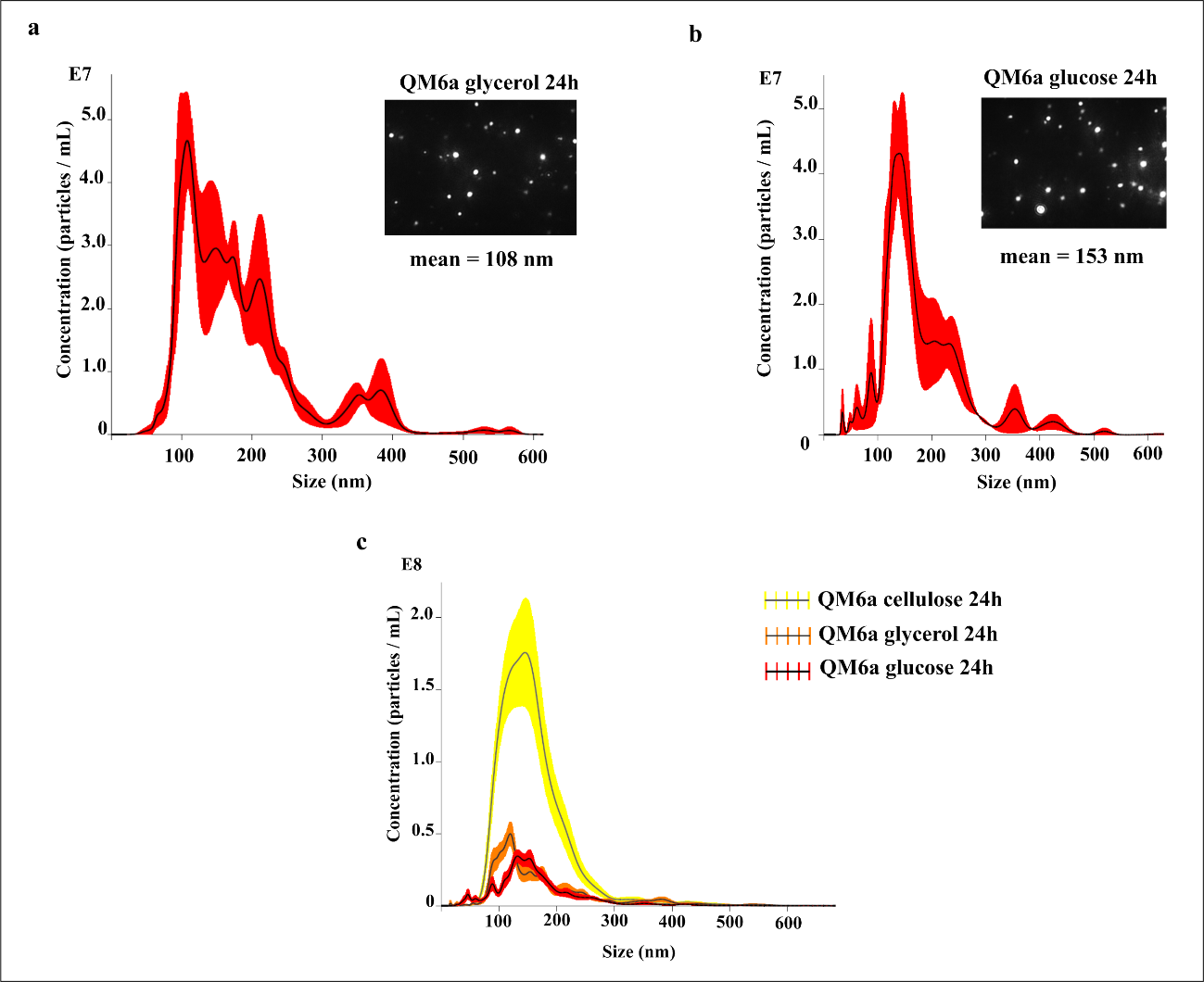


**Fig. S3** Characterization of *Trichoderma reesei* EVs. Mean size distribution by Nanoparticle Tracking Analysis (NTA) of purified *T. reesei* EVs from glycerol **(a)**, glucose **(b),** and both conditions **(c)** at 24 h. These results are based on three replicates of three independent experiments.


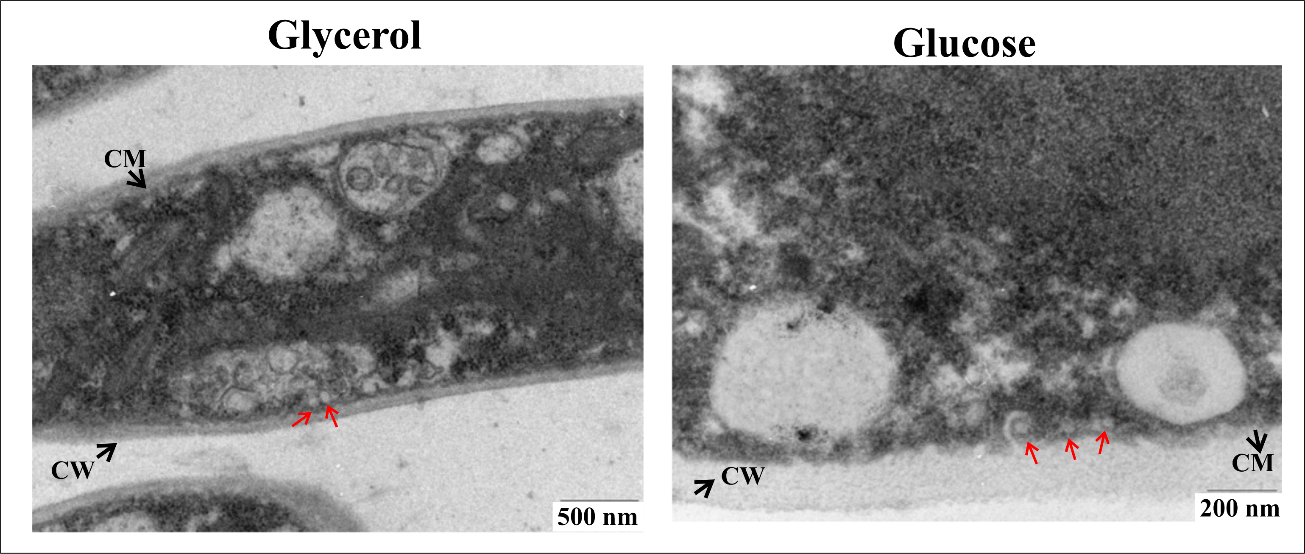


**Fig. S4** TEM analyzes of vesicles in *Trichoderma reesei* mycelium cells. The occurrence of vesicles in association with the cytoplasmic membrane and cell wall is evident after growth for 24 h in the presence of glucose and glycerol. Black arrows indicate the CW (cell wall) and CM (cell membrane). Red arrows indicate the *T. reesei* vesicles. Bars, 500 nm and 200 nm.

**
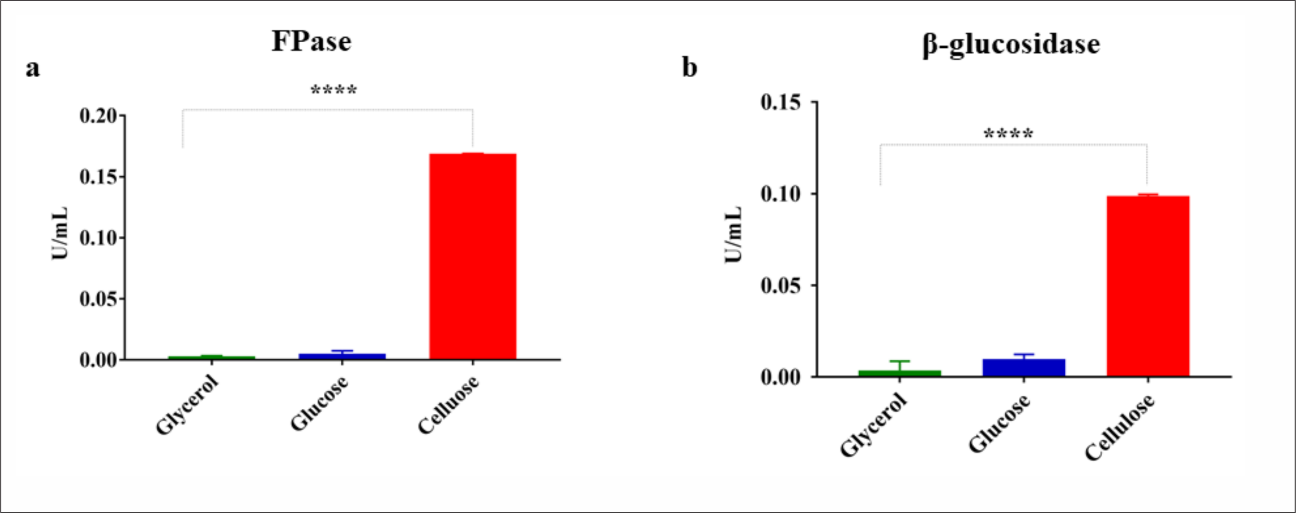
**

**Fig. S5** Cellulolytic activities from purified *T. reesei* EVs after growth in the presence of cellulose, glycerol and glucose. **(a)** Filter paper activity (FPase) and **(b)** β-glucosidase activity from purified *T. reesei* EVs grown at 24 h in the presence of respective carbon source. **** = Significantly different (P<0.001). These results are based on three replicates of three independent experiments and are expressed as mean ± standard deviation.
